# Protein intake and risk of frailty among older women in the Nurses' Health Study

**DOI:** 10.1002/jcsm.12972

**Published:** 2022-03-23

**Authors:** Ellen A. Struijk, Teresa T. Fung, Fernando Rodríguez‐Artalejo, Heike A. Bischoff‐Ferrari, Frank B. Hu, Walter C. Willett, Esther Lopez‐Garcia

**Affiliations:** ^1^ Department of Preventive Medicine and Public Health, School of Medicine Universidad Autónoma de Madrid‐IdiPaz Madrid Spain; ^2^ CIBERESP (CIBER of Epidemiology and Public Health) Madrid Spain; ^3^ Department of Nutrition Simmons University Boston MA USA; ^4^ Department of Nutrition Harvard T.H. Chan School of Public Health Boston MA USA; ^5^ IMDEA/Food Institute, CEI UAM+CSIC Madrid Spain; ^6^ Department of Geriatrics and Aging Research University Hospital Zurich and University of Zurich Zurich Switzerland; ^7^ Centre on Aging and Mobility University Hospital Zurich and Waid City Hospital Zurich Switzerland; ^8^ Channing Division of Network Medicine, Department of Medicine Brigham and Women's Hospital and Harvard Medical School Boston MA USA

**Keywords:** Protein, Diet, Frailty, Elderly

## Abstract

**Background:**

There is evidence that an overall healthy diet is associated with lower risk of frailty. However, the effect of diet composition, specifically the role of protein intake on frailty, is mostly unclear. The aim of this study was to evaluate the intake of protein, including total, plant, animal, and dairy protein, in relation to frailty incidence in a large cohort of older women.

**Methods:**

We analysed data from 85 871 women aged ≥60 participating in the Nurses' Health Study. Intake of protein was measured nine times during follow‐up from 1980 until 2010. Frailty was defined as having at least three of the following five criteria from the Fatigue, Resistance, Ambulation, Illnesses and Loss of Weight (FRAIL) scale: fatigue, low strength, reduced aerobic capacity, having ≥5 illnesses, and weight loss of ≥5%. The occurrence of frailty was assessed every 4 years from 1992 up to 2014.

**Results:**

During follow‐up, we identified 13 279 incident cases of frailty. Women with a higher intake of plant protein had a lower risk of developing frailty after adjustment for all relevant confounders [relative risks across quintiles of consumption: 1.00, 0.94, 0.89, 0.86, and 0.86; *P*‐trend < 0.001]. In contrast, those with a higher intake of animal protein intake had a higher risk of frailty [relative risks across quintiles of consumption: 1.00, 0.98, 0.99, 1.00, and 1.07; *P*‐trend 0.04]. The intake of total and dairy protein showed no significant association with frailty in the full model. Substituting 5% of energy from plant protein intake at the expense of animal protein, dairy protein, or non‐dairy animal protein was associated with 38% (29%, 47%), 32% (21%, 42%), and 42% (33%, 50%) reduced risk of frailty.

**Conclusions:**

A higher intake of plant protein, but not animal or dairy protein, was associated with a lower risk of frailty. Substitution of plant protein for animal protein, especially non‐dairy animal protein, was associated with lower risk of frailty.

## Introduction

The syndrome of frailty has been defined as a state of increased vulnerability to health stressors. The development of frailty is progressive, with severe dysregulation of key biological systems, leading to sarcopenia and malnutrition, as well as to weakness and comorbidity.^1^ Therefore, frail older adults have a higher risk of falls, disability, hospitalization, and death.^2^ Because frailty places a huge burden on public health in an aging society, it is important to identify the determinants of frailty.

Overall diet quality has previously been linked to the risk of frailty.^3^, ^4^, ^5^ Among dietary components, protein intake is thought to play an important role, because a deficient intake has a detrimental effect on muscle mass and strength.^6^, ^7^ Reduced total protein intake has also been associated with lower levels of appendicular lean mass, adjusted for body size,^8^ an optimal measure to define both sarcopenia and malnutrition.^9^ Moreover, protein intake above the current recommended dietary allowance of 0.8 g protein/kg/day^10^ has shown to reduce hip fractures and bone mass density loss^11^, ^12^, ^13^ and helps maintain physical function.^14^

Proteins from different sources vary in amino acid profiles, which may have different effects on muscle protein synthesis. Specifically, ‘fast’ proteins such as whey protein are rapidly digested and absorbed and may therefore have a great impact on muscle protein accretion.^15^ Leucine, an amino acid mainly present in animal products, is suggested to have a positive effect on signalling pathways for muscle protein synthesis, sarcopenia, and, possibly, frailty.^16^, ^17^ Also, previous research has found that increased intake of plant protein, but not animal protein, has been associated with delayed aging and better health, but it is unclear if this extends to a lower risk of frailty.^18^

Although the current literature suggests that older adults are at increased need for protein, it is not well established whether an adequate protein intake prevents frailty and whether different types of proteins have a different effect on this outcome.^19^ Thus, we investigated the association of habitual protein intake, including total, plant, animal, and dairy protein, with the risk of frailty in a large cohort of older women from the Nurses' Health Study (NHS).

## Methods

### Study design and participants

The NHS was established in 1976 with the enrolment of 121 700 female nurses aged 30 to 55 years at inception.^20^ Participants completed biennial mailed questionnaires to update information on medical history and lifestyle. The follow‐up rate was approximately 90% at each follow‐up cycle. The Harvard T.H. Chan School of Public Health and the Brigham and Women's Hospital Human Subjects Committee Review Board approved the protocol for the study.

For this analysis, we included women aged ≥60 years at baseline with complete information on the exposure and outcome variables. Women younger than 60 years at baseline entered the study when they turned 60 during follow‐up. Women with an unreasonably high (>3500 kcal/day) or low (<500 kcal/day) energy intake were excluded from follow‐up, as well as women identified as frail at analytical baseline, leaving a total population of 85 871 women for analysis. The relationship between protein intake and frailty occurrence was examined up to 2014.

### Dietary assessment

Dietary intake was measured using a validated food frequency questionnaire in 1980, 1984, 1986, 1990, 1994, 1998, 2000, 2006, and 2010 as described in detail elsewhere.^21^ In each questionnaire, participants were asked how often on average during the previous year they had consumed the foods specified. A standard portion size and nine possible responses for the frequency of consumption ranging from ‘never, or less than once per month’ to ‘6 or more times per day’ were given for each food item. Nutrient and energy intakes were calculated by multiplying the consumption of each food recorded by its nutrient and energy content, using the US Department of Agriculture database and complemented with information from the manufacturers. Intakes of plant, animal (including dairy), and dairy protein were calculated for each participant and expressed as a percentage of total energy by multiplying the grams of protein consumed per day by the number of kilocalories in 1 g of protein (4 kcal/g) and then divided by total energy intake. We used the percentage of energy from each macronutrient instead of absolute intake to reduce bias owing to underreporting of food consumption and to represent dietary composition.^22^ Main food sources of plant protein were bread, cereals, pasta, nuts, beans, and legumes; main food sources of animal protein included processed and unprocessed red meat, poultry, fish and seafood, eggs, and dairy products. Previous research showed that the food frequency questionnaire is reasonably valid and consistent for measuring nutrient intakes compared with multiple dietary records, 24 h dietary recalls, and biomarkers of diet among women.^21^, ^23^ The validation studies revealed good correlations of protein intake assessed by the food frequency questionnaire with multiple dietary records (*r* = 0.54) and its biomarker (*r* = 0.46).

To best represent long‐term diet during follow‐up and to account for changes in food consumption, we used the cumulative average of protein intake from all available dietary questionnaires from 1980 through frailty onset or the end of follow‐up. In addition, a modified Alternate Healthy Eating Index (AHEI) score was used as an indicator of overall diet quality. This score was calculated based on 10 foods and nutrients that are beneficial reducing chronic disease risk, including high consumption of fruit, vegetables, nuts, legumes, and whole grains, high intake of long chain omega‐3 and other polyunsaturated fats, moderate consumption of alcohol, and low consumption of red and processed meat, sodium, *trans* fat, and sugar‐sweetened beverages.^24^ A higher score in the AHEI denotes better diet quality.

### Frailty assessment

Frailty was assessed by the Fatigue, Resistance, Ambulation, Illnesses and Loss of Weight (FRAIL) scale^25^ that includes five self‐reported frailty criteria: fatigue, low strength (reduced resistance), reduced aerobic capacity, having several chronic illnesses, and recent significant unintentional weight loss. In 1992, 1996, 2000, 2004, 2008, and 2012, NHS participants completed the Medical Outcomes Study Short‐Form (SF‐36), a 36‐item questionnaire with eight health dimensions, including physical and mental components.^26^ From the SF‐36, we assessed the first three frailty criteria with the following questions: (i) for fatigue: ‘Did you have a lot of energy?’, with response options ‘some of the time’ or ‘none of the time’ (in years 1992, 1996, and 2000), or with the question ‘I could not get going’ in an updated version of the SF‐36 (in 2004, 2008, and 2012), with response options ‘moderate amount’ or ‘all of the time’; (ii) for low strength: ‘In a normal day, is your health a limitation to walk up 1 flight of stairs?’, with response options ‘yes’ or ‘a lot’; and (iii) for reduced aerobic capacity: ‘In a normal day, is your health a limitation to walk several blocks or several miles?’, with response options ‘yes’ or ‘a lot’. In addition, the illnesses criterion was assessed from the question, ‘In the last 2 years, have you had any of these physician‐diagnosed illnesses?’. We considered that this criterion was met when participants reported ≥5 of the following diseases: cancer, hypertension, type 2 diabetes, angina, myocardial infarction, stroke, congestive heart failure, asthma, chronic obstructive lung disease, arthritis, Parkinson's disease, kidney disease, and depression. Finally, the weight loss criterion was defined as a ≥5% decrease in the weight reported in a 2 year period. At the end of each follow‐up cycle, incident frailty was defined as having ≥3 criteria in the FRAIL scale. The FRAIL scale has been shown to be correlated (*r* = 0.62, *P* < 0.001) with the Physical Frailty Phenotype,^27^ the most widely used scale for frailty assessment, which includes both self‐reported and performance‐based measures.

### Ascertainment of mortality

Deaths were reported by next of kin, or the postal system, or ascertained through the National Death Index. Follow‐up for mortality was more than 98% complete.^28^ We obtained copies of death certificates and medical records to determine causes of death (classified according to the categories of the International Classification of Diseases, Ninth Revision). Death records were reviewed and coded by physicians.

### Medical history, anthropometric data, and lifestyle factors

In the analytic baseline questionnaire, we collected information on age, weight, smoking status, and medication use. This information has been updated on each of the subsequent biennial questionnaires. To calculate body mass index (BMI), we used information on height measured in 1976, when the cohort was initiated; BMI was calculated as weight in kilograms divided by the square of height in metres. Discretionary physical activity was reported as the average time spent per week during the preceding year in specific activities (e.g. walking outdoors, jogging, and bicycling). The time spent in each activity was multiplied by its typical energy expenditure, expressed in metabolic equivalent tasks, and then summed over all activities.

### Statistical analysis

Participants were classified into five groups according to quintiles of percentage of energy from protein intake. We used Cox proportional hazards models to calculate relative risks (RRs), approximated by hazard ratios, and their 95% confidence interval (CI) for the association between protein intake and frailty, adjusting for potential confounders updated at each 4 year cycle. Person‐years were calculated from baseline until the occurrence of frailty, death, or the end of the study period (1 June 2014), whichever came first. Multivariable models were adjusted for baseline BMI, smoking status, alcohol intake, energy intake, and medication use. Results were further adjusted for percentages of energy derived from saturated fat, monounsaturated fat, polyunsaturated fat, *trans* fat, and dietary cholesterol. An additional model was built with adjustment for diet quality using the AHEI. All models included mutual adjustment for percentages of energy derived from each type of protein (quintiles): plant protein models were adjusted for animal protein and vice versa, and dairy protein models were adjusted for non‐dairy animal protein and plant protein. Because physical activity is closely related to the outcome, adjustment for baseline physical activity was only done in secondary analyses. Tests for linear trend were conducted by assigning the median value to each quintile and treating this as a continuous variable in the regression models. We also modelled the risk of frailty for each 5% of energy intake of protein and assessed the association between protein intake and each criterion of the FRAIL scale.

With substitution analysis, we estimated the effect of substituting one type of protein for an equal exchange of another type of protein on frailty risk. To fit these models, we simultaneously included total energy intake and the percentage of energy derived from the types of protein of interest as continuous variables, along with the covariates listed above.

Several sensitivity analyses were performed. We stratified the association between protein intake and frailty by baseline BMI (<25 kg/m^2^ compared with ≥25 kg/m^2^), physical activity (above or below the median), AHEI (above or below the median score), and baseline age (<70 years compared with ≥70 years).^29^ Interaction was evaluated using the Wald test on cross‐product terms based on protein intake (continuous variable) and the stratification variable. We also replicated the analyses among those with none of the frailty criteria at baseline, and using a stricter definition of frailty requiring four instead of three frailty criteria to define someone as frail. Additionally, to assess bias caused by the possibility that women with early signs of frailty may have changed their diet, 8 year lagged analyses were conducted. Simple updated analysis using the most recent protein intake was conducted to assess the shorter‐term effect of this intake on frailty. All statistical tests were two‐sided with a *P* value < 0.05 and performed using SAS software, Version 9.4 for UNIX (SAS Institute Inc, Cary, NC).

## Results

The average (standard deviation) total protein intake among the 85 871 women in the study was 18.31 (2.55) % of energy. Animal protein was the main source of protein in this population (13.27 ± 2.68) followed by plant protein (5.05 ± 0.90) and dairy protein (3.77 ± 1.57). *Table* 1 shows the baseline characteristics of participants according to the intake of the different types of protein. Compared with participants in the lowest quintile of plant protein intake, those in the highest quintile had a lower BMI, were less often current smokers, were more physically active, and adhered to a healthier diet as measured with the AHEI. In contrast, compared with participants in the lowest quintile of animal protein intake, those in the highest quintile had a higher BMI, but were also less often smokers and adhered to a healthier diet. The trends were less clear for dairy protein, but those with the highest dairy protein intake showed a higher BMI, were also less often smokers and more physically active, and adhered to a healthier diet compared with those with a lower intake.

**Table 1:** Baseline characteristics according to quintiles of protein intake (% of energy) among women aged ≥60 years in the Nurses' Health Study

	Plant protein			Animal protein			Dairy protein		
	Q1	Q3	Q5	Q1	Q3	Q5	Q1	Q3	Q5
Range	0.92–3.99	4.44–4.84	5.34–13.3	0.24–11.6	13.2–14.6	16.4–39.7	0.01–2.36	3.21–4.06	5.22–20.3
Participants, *n*	18 991	17 060	15 468	15 792	17 432	17 906	17 487	17 508	15 932
Mean age, years	62.4 (2.2)	62.6 (2.2)	62.8 (2.4)**	62.7 (2.4)	62.6 (2.3)	62.5 (2.2)**	62.6 (2.3)	62.6 (2.2)	62.8 (2.4)**
BMI, kg/m^2^	26.0 (5.0)	25.8 (4.7)	25.0 (4.6)**	24.5 (4.4)	25.5 (4.6)	27.0 (5.1)**	25.3 (4.8)	25.8 (4.7)	25.9 (4.8)**
Current smoker, %	18	11	8**	15	12	11**	16	11	10**
Discretionary physical activity, METs‐h/week	15.8 (21.3)	18.7 (22.3)	23.2 (27.1)**	18.9 (23.1)	18.7 (22.9)	19.0 (23.4)**	17.1 (22.1)	19.3 (24.2)	20.1 (23.4)**
Medication use^a^									
Aspirin, %	41	47	46**	45	47	44**	43	47	46**
Postmenopausal hormone therapy, %	35	38	39**	35	38	38**	36	37	38
Diuretics, %	9	11	9	8	10	11**	10	10	9
Beta‐blockers, %	13	13	12	12	13	14**	13	13	12
Calcium channel blockers, %	9	10	10**	9	10	11**	10	10	9
ACE inhibitors, %	10	10	9	8	10	11**	10	10	10
Other blood pressure medication, %	9	9	8*	8	8	9**	9	9	8
Statins, %	13	15	15**	13	14	16**	15	15	14*
Other cholesterol lowering drugs, %	3	3	3	3	3	4**	3	4	4
Insulin, %	1	2	2**	1	1	3**	1	1	2**
Oral hypoglycaemic drugs, %	3	3	3	2	3	4**	3	3	3**
Number of frailty criteria, %									
0	73	74	77**	76	75	72**	74	74	74**
1	22	21	19**	20	21	22**	21	21	21**
2	6	5	4**	4	5	6**	5	5	5**
Dietary intake									
Energy intake, kcal/day	1728 (452)	1720 (421)	1663 (428)**	1809 (465)	1737 (414)	1561 (397)**	1656 (446)	1730 (430)	1704 (412)**
Total protein (% of energy)	18.4 (3.1)	18.6 (2.5)	18.7 (2.7)**	15.2 (1.5)	18.3 (0.9)	22.2 (2.0)**	17.4 (2.8)	18.4 (2.4)	20.2 (2.6)**
Plant protein (% of energy)	3.8 (0.5)	4.8 (0.2)	6.2 (0.7)**	5.2 (1.1)	4.8 (0.8)	4.5 (0.8)**	4.9 (1.0)	4.9 (0.8)	4.7 (0.9)**
Animal protein (% of energy)	14.6 (3.1)	13.8 (2.5)	12.6 (2.9)**	10.0 (1.4)	13.5 (0.6)	17.7 (2.0)**	12.6 (2.9)	13.5 (2.5)	15.5 (2.8)**
Dairy protein (% of energy)	3.9 (1.9)	3.8 (1.5)	3.5 (1.5)**	2.9 (1.2)	3.7 (1.4)	4.6 (2.0)**	1.8 (0.5)	3.6 (0.2)	6.3 (1.2)**
Total fat (% of energy)	34.3 (6.5)	32.1 (5.3)	29.3 (5.7)**	31.0 (6.0)	32.3 (5.6)	32.4 (6.3)**	33.1 (6.3)	32.2 (5.7)	30.5 (5.7)**
Saturated fat (% of energy)	13.1 (2.9)	11.5 (2.1)	9.8 (2.2)**	10.9 (2.6)	11.7 (2.4)	11.9 (2.8)**	11.4 (2.7)	11.6 (2.5)	11.6 (2.7)**
Polyunsaturated fat (% of energy)	5.3 (1.3)	5.6 (1.2)	5.8 (1.4)**	5.8 (1.4)	5.6 (1.2)	5.4 (1.2)**	5.9 (1.4)	5.6 (1.2)	5.1 (1.1)**
Carbohydrates (% of energy)	43.3 (7.7)	47.4 (5.5)	51.3 (6.2)**	51.6 (6.9)	47.1 (5.8)	43.4 (6.5)**	46.2 (7.9)	47.2 (6.4)	48.2 (6.3)**
Alcohol intake, (g/day)	8.7 (12.4)	5.5 (7.6)	3.9 (6.0)**	7.2 (11.4)	6.3 (8.9)	4.3 (6.5)**	7.8 (11.4)	6.0 (8.5)	4.1 (6.7)**
AHEI score	45.8 (8.2)	52.1 (8.0)	60.0 (8.8)**	50.5 (10.4)	51.6 (9.1)	55.0 (9.0)	50.6 (9.9)	52.5 (9.3)	53.3 (9.4)**

ACE, angiotensin‐converting enzyme; AHEI, Alternate Healthy Eating Index; BMI, body mass index; METs, metabolic equivalent tasks.

Values are means (standard deviation) unless otherwise indicated. Data, except age, were directly standardized to the age distribution of the entire cohort.

^a^ One or more times per week.

* *P* value < 0.01.

** *P* value < 0.001.

During over 22 years of follow‐up, we identified a total of 13 279 incident frailty cases (*Table* 2). Women with a higher total protein intake had a lower risk of developing frailty after adjustment for lifestyle, medication use, and dietary fat intake [RRs (95% CI) across quintiles of consumption: 1.00, 0.93 (0.88–0.99), 0.92 (0.87–0.98), 0.89 (0.84–0.95), and 0.93 (0.88–0.99); *P*‐trend 0.03]; however, this association attenuated and changed to detrimental for those in Quintile 5, after further adjustment for diet quality [RRs (95% CI) across quintiles of intake: 1.00, 0.97 (0.91–1.02), 0.98 (0.93–1.04), 0.97 (0.91–1.03), and 1.06 (0.99–1.13); *P*‐trend 0.06]. A higher intake of plant protein was associated with lower risk of frailty in a dose–response manner [RRs (95% CI) across quintiles of intake: 1.00, 0.94 (0.89–0.99), 0.89 (0.84–0.94), 0.86 (0.81–0.91), and 0.86 (0.80–0.92); *P*‐trend < 0.001] in the full model. In contrast, those in the highest quintile of animal protein intake had a higher risk of frailty after adjusting for main confounders [RRs (95% CI) across quintiles of intake: 1.00, 0.98 (0.93–1.04), 0.99 (0.93–1.05), 1.00 (0.94–1.06), and 1.07 (1.00–1.14); *P*‐trend 0.04]. Lastly, dairy protein was not associated with risk of frailty in the full model [RRs (95% CI) across quintiles of intake: 1.00, 1.00 (0.95–1.06), 0.99 (0.94–1.05), 1.03 (0.97–1.09), and 1.02 (0.96–1.08); *P*‐trend 0.33]. When physical activity was additionally included in the model, the estimates changed only marginally (Supporting Information, *Table* S1).

**Table 2:** Relative risks (95% confidence interval) of frailty according to quintiles of protein intake (% of energy) among 85 871 women aged ≥60 years in the Nurses' Health Study

	Q1	Q2	Q3	Q4	Q5	*P* for trend
**Total protein**						
Participants, *n*	16 478	17 209	17 374	17 474	17 336	
Person‐years	244 455	245 090	244 907	244 795	243 427	
Frailty events, *n*	2624	2487	2567	2593	3008	
Age‐adjusted	1.00	0.99 (0.94; 1.05)	1.04 (0.99; 1.10)	1.07 (1.01; 1.13)	1.26 (1.20; 1.33)	<0.001
Model 1^a^	1.00	0.94 (0.89; 1.00)	0.94 (0.89; 0.99)	0.91 (0.86; 0.96)	0.95 (0.90; 1.00)	0.06
Model 2^b^	1.00	0.93 (0.88; 0.99)	0.92 (0.87; 0.98)	0.89 (0.84; 0.95)	0.93 (0.88; 0.99)	0.03
Model 3^c^	1.00	0.97 (0.91; 1.02)	0.98 (0.93; 1.04)	0.97 (0.91; 1.03)	1.06 (0.99; 1.13)	0.06
**Plant protein**						
Participants, *n*	18 991	17 833	17 060	16 519	15 468	
Person‐years	241 420	244 201	245 126	245 667	246 259	
Frailty events, *n*	2886	2796	2674	2516	2407	
Age‐adjusted	1.00	0.92 (0.87; 0.97)	0.84 (0.80; 0.89)	0.77 (0.73; 0.82)	0.70 (0.66; 0.74)	<0.001
Model 1^a^	1.00	0.91 (0.87; 0.96)	0.84 (0.79; 0.88)	0.77 (0.73; 0.82)	0.72 (0.68; 0.76)	<0.001
Model 2^b^	1.00	0.91 (0.86; 0.96)	0.83 (0.79; 0.88)	0.77 (0.73; 0.82)	0.73 (0.68; 0.78)	<0.001
Model 3^c^	1.00	0.94 (0.89; 0.99)	0.89 (0.84; 0.94)	0.86 (0.81; 0.91)	0.86 (0.80; 0.92)	<0.001
**Animal protein**						
Participants, *n*	15 792	17 124	17 432	17 617	17 906	
Person‐years	245 175	245 352	245 021	244 431	242 694	
Frailty events, *n*	2521	2474	2553	2696	3035	
Age‐adjusted	1.00	1.05 (0.99; 1.11)	1.12 (1.06; 1.19)	1.21 (1.14; 1.27)	1.41 (1.34; 1.49)	<0.001
Model 1^a^	1.00	1.00 (0.95; 1.06)	1.00 (0.95; 1.06)	1.01 (0.95; 1.07)	1.06 (1.00; 1.12)	0.04
Model 2^b^	1.00	0.97 (0.91; 1.02)	0.96 (0.90; 1.02)	0.95 (0.89; 1.01)	0.97 (0.91; 1.04)	0.41
Model 3^c^	1.00	0.98 (0.93; 1.04)	0.99 (0.93; 1.05)	1.00 (0.94; 1.06)	1.07 (1.00; 1.14)	0.04
**Dairy protein**						
Participants, *n*	17 487	17 833	17 508	17 111	15 932	
Person‐years	244 042	244 828	245 003	244 694	244 107	
Frailty events, *n*	2529	2542	2563	2720	2925	
Age‐adjusted	1.00	1.02 (0.96; 1.08)	1.01 (0.96; 1.07)	1.05 (1.00; 1.11)	1.07 (1.01; 1.13)	0.01
Model 1^a^	1.00	1.00 (0.94; 1.05)	0.98 (0.92; 1.03)	1.01 (0.95; 1.06)	1.00 (0.95; 1.06)	0.85
Model 2^b^	1.00	0.98 (0.92; 1.03)	0.95 (0.90; 1.01)	0.97 (0.91; 1.03)	0.95 (0.89; 1.00)	0.09
Model 3^c^	1.00	1.00 (0.95; 1.06)	0.99 (0.94; 1.05)	1.03 (0.97; 1.09)	1.02 (0.96; 1.08)	0.33

^a^ Adjusted for age (months), calendar time (4 year interval), baseline body mass index (<25.0, 25.0–29.9, and ≥30.0 kg/m^2^), smoking status (never, past, and current 1–14, 15–24, and ≥25 cigarettes/day), alcohol intake (0, 1.0–4.9, 5.0–14.9, or ≥15.0 g/day), energy intake (quintiles of kcal/day), and medication use (aspirin, postmenopausal hormone therapy, diuretics, beta‐blockers, calcium channel blockers, angiotensin‐converting enzyme inhibitors, other blood pressure medication, statins and other cholesterol lowering drugs, insulin, and oral hypoglycaemic medication).

^b^ Adjusted for variables in Model 1 and additionally adjusted for percentages of energy from saturated fat, monounsaturated fat, polyunsaturated fat, *trans* fat, and dietary cholesterol (all in quintiles).

^c^ Adjusted for variables in Model 2 and additionally adjusted for the Alternate Healthy Eating Index (quartiles).

Plant protein models were adjusted for animal protein and vice versa. Dairy protein models were adjusted for non‐dairy animal protein and plant protein.

**Figure 1:**
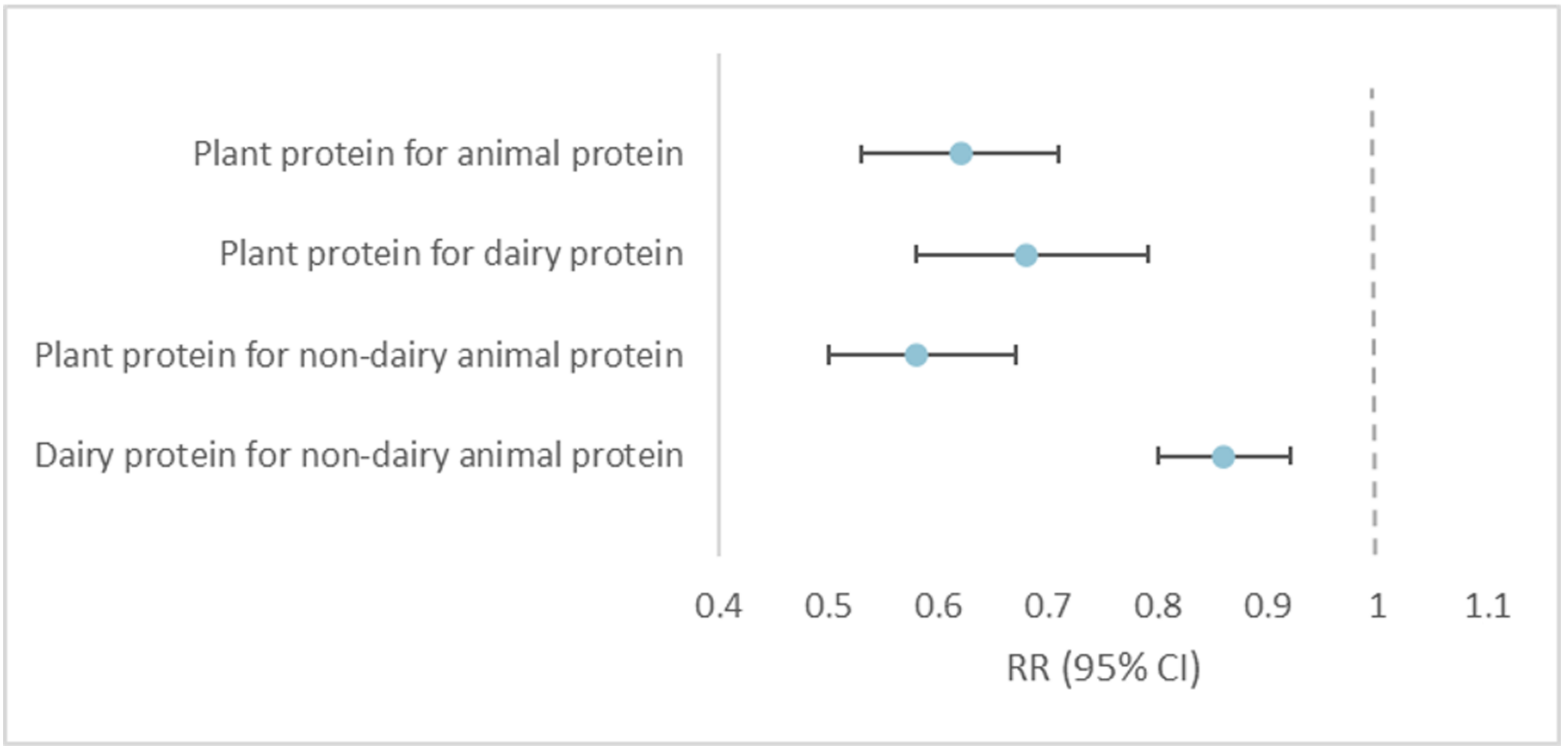
Relative risks (95% CI) of frailty for substitution of 5% of energy from protein for equal exchanges of other types of protein among women aged ≥60 years in the Nurses' Health Study. Adjusted for age (months), calendar time (4 year interval), body mass index (<25.0, 25.0–29.9, and ≥30.0 kg/m^2^), smoking status (never, past, and current 1–14, 15–24, and ≥25 cigarettes/day), alcohol intake (0, 1.0–4.9, 5.0–14.9, or ≥15.0 g/day), energy intake (quintiles of kcal/day) and medication use (aspirin, postmenopausal hormone therapy, diuretics, beta‐blockers, calcium channel blockers, angiotensin‐converting enzyme inhibitors, other blood pressure medication, statins and other cholesterol lowering drugs, insulin, and oral hypoglycaemic medication), percentages of energy from saturated fat, monounsaturated fat, polyunsaturated fat, *trans* fat, and dietary cholesterol (all in quintiles), and the Alternate Healthy Eating Index (quartiles). CI, confidence interval; RR, relative risk.

Substituting 5% of energy from plant protein intake at the expense of animal protein, dairy protein, or non‐dairy animal protein (including protein from meat, fish, and eggs) was associated with 38% (29–47%), 32% (21–42%), and 42% (33–50%) reduced risk of frailty (*Figure* 1). Additionally, substituting dairy protein for non‐dairy animal protein was associated with a 14% lower risk of frailty (8–20%).

Total protein intake was associated with higher risk for the criteria of having low strength (RRs Q5 vs. Q1: 1.07; 95% CI: 1.00–1.14; *P*‐trend 0.02) and ≥5 illnesses (1.25; 95% CI: 1.13–1.39; *P*‐trend < 0.001) (*Table* 3). When examining specific types, an increase in plant protein intake showed an inverse association with low strength (0.82; 95% CI: 0.76–0.88; *P*‐trend < 0.001) and reduced aerobic capacity (0.89; 0.85–0.94; *P*‐trend < 0.001). For animal protein intake, a significant detrimental association was seen for the criteria of having low strength (1.09; 1.02–1.16; *P*‐trend 0.02) and ≥5 illnesses (1.35; 1.21–1.50; *P*‐trend < 0.001), but a significant protective effect for the weight loss criteria (0.96; 0.91–1.00; *P*‐trend 0.05). No associations were found between the intake of dairy protein and any of the frailty criteria.

**Table 3:** Relative risks (95% confidence interval) of frailty components according to quintiles of protein intake (% of energy) among 85 871women aged ≥60 years in the Nurses' Health Study

	Q1	Q2	Q3	Q4	Q5	*P* for trend
**Total protein**						
Fatigue	1.00	0.99 (0.95; 1.02)	1.01 (0.97; 1.04)	0.99 (0.96; 1.03)	1.01 (0.97; 1.05)	0.53
Low strength	1.00	0.97 (0.91; 1.03)	1.02 (0.96; 1.09)	1.01 (0.95; 1.08)	1.07 (1.00; 1.14)	0.02
Reduced aerobic capacity	1.00	0.96 (0.92; 1.00)	1.00 (0.96; 1.04)	0.99 (0.94; 1.03)	1.02 (0.97; 1.06)	0.30
≥5 illnesses	1.00	1.04 (0.95; 1.14)	1.06 (0.96; 1.17)	1.20 (1.08; 1.32)	1.25 (1.13; 1.39)	<0.001
Weight loss	1.00	1.01 (0.97; 1.04)	0.99 (0.95; 1.02)	0.99 (0.95; 1.03)	0.98 (0.94; 1.03)	0.33
**Plant protein**						
Fatigue	1.00	1.00 (0.96; 1.03)	0.80 (0.94; 1.01)	0.98 (0.94; 1.02)	1.00 (0.96; 1.05)	0.91
Low strength	1.00	0.89 (0.84; 0.95)	0.86 (0.81; 0.91)	0.81 (0.76; 0.87)	0.82 (0.76; 0.88)	<0.001
Reduced aerobic capacity	1.00	0.92 (0.89; 0.96)	0.91 (0.87; 0.95)	0.89 (0.85; 0.93)	0.89 (0.85; 0.94)	<0.001
≥5 illnesses	1.00	0.98 (0.89; 1.07)	0.93 (0.84; 1.02)	0.99 (0.90; 1.10)	0.97 (0.86; 1.09)	0.72
Weight loss	1.00	0.99 (0.96; 1.03)	0.98 (0.94; 1.02)	0.97 (0.93; 1.01)	0.97 (0.93; 1.02)	0.17
**Animal protein**						
Fatigue	1.00	0.99 (0.96; 1.03)	1.00 (0.97; 1.04)	1.01 (0.97; 1.05)	1.02 (0.98; 1.06)	0.34
Low strength	1.00	0.99 (0.94; 1.05)	1.04 (0.98; 1.11)	1.03 (0.97; 1.10)	1.09 (1.02; 1.16)	0.01
Reduced aerobic capacity	1.00	0.98 (0.94; 1.02)	1.01 (0.97; 1.05)	0.99 (0.95; 1.04)	1.03 (0.98; 1.07)	0.20
≥5 illnesses	1.00	1.16 (1.06; 1.28)	1.13 (1.02; 1.24)	1.27 (1.15; 1.40)	1.35 (1.21; 1.50)	<0.001
Weight loss	1.00	0.99 (0.96; 1.03)	0.95 (0.91; 0.99)	0.97 (0.93; 1.01)	0.96 (0.91; 1.00)	0.05
**Dairy protein**						
Fatigue	1.00	1.01 (0.98; 1.05)	1.02 (0.98; 1.05)	1.02 (0.98; 1.05)	1.00 (0.96; 1.03)	0.77
Low strength	1.00	0.96 (0.90; 1.02)	1.00 (0.94; 1.06)	1.02 (0.96; 1.08)	1.02 (0.96; 1.09)	0.14
Reduced aerobic capacity	1.00	0.98 (0.94; 1.02)	0.98 (0.94; 1.02)	1.00 (0.96; 1.04)	1.02 (0.97; 1.06)	0.21
≥5 illnesses	1.00	0.95 (0.86; 1.03)	1.00 (0.91; 1.10)	1.06 (0.97; 1.16)	1.02 (0.92; 1.12)	0.24
Weight loss	1.00	1.00 (0.96; 1.03)	0.99 (0.95; 1.03)	1.00 (0.96; 1.04)	0.99 (0.95; 1.04)	0.86

Adjusted for age (months), calendar time (4 year interval), baseline body mass index (<25.0, 25.0–29.9, and ≥30.0 kg/m^2^), smoking status (never, past, and current 1–14, 15–24, and ≥25 cigarettes/day), alcohol intake (0, 1.0–4.9, 5.0–14.9, or ≥15.0 g/day), energy intake (quintiles of kcal/day), medication use (aspirin, postmenopausal hormone therapy, diuretics, beta‐blockers, calcium channel blockers, angiotensin‐converting enzyme inhibitors, other blood pressure medication, statins and other cholesterol lowering drugs, insulin, and oral hypoglycaemic medication), percentages of energy from saturated fat, monounsaturated fat, polyunsaturated fat, *trans* fat, and dietary cholesterol (all in quintiles), and the Alternate Healthy Eating Index (quartiles). Plant protein models were adjusted for animal protein and vice versa. Dairy protein models were adjusted for non‐dairy animal protein and plant protein.

In stratified analyses, a significant interaction was found between total and animal protein intake and BMI; the association among women with a BMI ≥ 25 kg/m^2^ was significantly detrimental, while this was non‐significant among those with BMI < 25 kg/m^2^ (*P* value = 0.004 and 0.01) (*Table* S2). Additionally, a significant interaction was found between plant protein intake and diet quality (*P* value = 0.03). Among women with a low diet quality, a high plant protein intake was inversely associated with frailty more strongly than among those with better diet quality [RRs per continuous % energy (95% CI): 0.53; 0.43–0.64 vs. 0.71; 0.58–0.86; *P* value = 0.03]. Results did not vary across other subgroups.

When we only included participants without any frailty criteria at baseline or when we used a stricter frailty definition, the detrimental association between animal protein and frailty attenuated (*Tables* S3 and S4). The 8 year lagged analysis strengthened the positive association between total protein and animal protein intake and frailty (*Table* S5), while analyses using the most recent protein intake showed a significant inverse association for both total protein and animal protein (*Table* S6).

## Discussion

In this large cohort study, we found that women with a higher intake of plant protein had a lower risk of frailty. Habitual long‐term intake of total protein, animal protein, and dairy protein was not associated with lower risk of frailty. In addition, substitution of 5% of energy intake from plant protein for an equal exchange of animal protein, dairy protein, or non‐dairy animal protein was associated with a reduced risk of frailty.

Several studies have previously assessed the association of amount and quality of protein intake and frailty, with inconsistent results.^19^ Moreover, among them, only two studies had a prospective design and investigated plant protein and animal protein separately. In the Women's Health Initiative Observational Study, among 24 417 older women, those with a 20% higher total protein intake had a 12% lower risk of frailty after 3 years of follow‐up, which was independent of the source of protein.^30^ A Spanish cohort comprising 1822 older adults of the Seniors‐ENRICA study showed that those with a higher intake of total and animal protein, but not plant protein, had a strong reduced risk of frailty after 3.5 years of follow‐up.^31^ In addition, a recent prospective study using information from the Framingham Third Generation Study showed that total protein intake was associated with higher levels of appendicular lean mass and quadriceps strength, but the different types of proteins did not, suggesting that if the recommended protein intake is fulfilled, the source of protein may not contribute to musculoskeletal outcomes related to sarcopenia and malnutrition.^8^ The inconsistencies seen in the literature are possibly caused by several factors including the length of follow‐up, design of the study, age of the participants, degree of confounder adjustment, and, possibly, the definition of frailty used.

Previous studies examining the effect of protein intakes on various outcomes have reported inconsistent results for plant protein vs. animal protein. For example, plant protein has been associated with lower risk of hip fracture,^13^ type 2 diabetes,^32^ unhealthy aging,^18^ and cardiovascular and all‐cause mortality.^29^ In contrast, but in line with our results for the illnesses criteria of frailty, animal protein intake has been related to higher risk of main chronic diseases including type 2 diabetes,^32^ ischaemic heart disease,^33^ and mortality.^29^, ^34^, ^35^ These associations could be partially mediated by BMI.^32^ Despite controlling for different types of fats and diet quality, we cannot exclude that other component consumed simultaneously with plant protein and animal protein (including specific vitamins, fibre, sodium, and nitrites) contributed to the associations found. In fact, when we removed the effect of the types of fats in the models, the significant detrimental effect of animal protein on frailty disappeared. In addition, when we used a stricter frailty definition or only participants with no frailty criteria at baseline, the association between animal protein and frailty attenuated; however, this is possibly due to a reduction of power.

The possible different effects of plant protein vs. animal protein may be explained by its amino acid profile. For example, animal protein is high in leucine concentration, an essential amino acid thought to play an important role in muscle protein synthesis.^36^ Animal protein is also rich in creatine, which is synthesized from the amino acids glycine and arginine, and is suggested to increase muscle mass, strength, and functioning.^37^ Our analysis using the habitual long‐term intake of animal protein showed a positive association with frailty that disappeared after adjustment for different types of fat and diet quality. However, this association was significantly detrimental in the latency analysis, which discards the first 8 years of follow‐up. In contrast, analyses using the most recent animal protein intake showed a significant inverse association with frailty incidence, in line with studies with a short follow‐up that reported protective effects of animal intake on frailty incidence.^30^, ^31^ This suggests that among older women, animal protein intake has a short‐term protective effect on the risk of frailty. Thus, it is possible that the loss of muscle mass, which may occur at an earlier stage in frailty development, is limited due to the intake of animal protein. Over the longer term, age‐related chronic diseases may be a more important driver of frailty, so habitual intake of plant protein may become more relevant on reducing the risk. However, results with shorter difference in time between diet assessment and the measurement of the outcome need to be interpreted with caution because reverse confounding might play an important role.

Regarding dairy products, casein and whey are high‐quality proteins rich in branched‐chain amino acids that have also shown to promote muscle protein synthesis. Specifically, whey protein seems to improve muscle protein anabolism due to its fast rates of digestion and absorption.^15^ However, in our study, dairy protein did not show a significant effect on frailty or its components after adjustment for diet quality; further research is needed on this finding. Lastly, some evidence suggests that amino acids act synergistically with exercise to increase fractional protein synthesis due to its impact on the mTORC1 signalling pathway in human skeletal muscle, which stimulates muscle protein synthesis and consequently prevents muscle loss.^15^, ^38^ However, in our sensitivity analysis, no clear difference was seen in the association between protein intake and frailty in women with a high physical activity compared with those with a lower physical activity.

A major strength of this study is the large sample size and the use of repeated dietary assessments and updated information on covariates over more than 22 years of follow‐up. However, several limitations need to be acknowledged. First, because dietary information was self‐reported, measurement error and misclassification could occur. However, the food frequency questionnaire used has been extensively validated against diet records and biomarkers, showing a good correlation for protein intake.^21^, ^23^ Second, although we were able to adjust for many potential confounders, residual and unmeasured confounding cannot be completely ruled out. Third, only one definition of frailty was used; our results should be confirmed in studies using other definitions, including the Physical Frailty Phenotype, the Deficit Accumulation Index, and the Vulnerable Elders Survey, the most common instruments in clinical and research settings,^27^, ^39^ or new and easier instruments, such as the Simpler Modified Fried Frailty Scale,^40^ or the SARC‐F questionnaire.^41^ Finally, although lagged analyses showed a consistent protective association between plant protein intake and frailty, the possibility of reverse causation cannot be totally discarded; individuals becoming frail may develop alterations in the metabolic system that lead to decreased appetite and subsequent changes in dietary habits, which could accelerate the progression to frailty.

In conclusion, among older adults, a higher intake of plant protein was associated with reduced risk of frailty. Moreover, these data suggest that replacing animal protein with plant protein might help to avoid the development of the frailty syndrome.

## Conflict of interest

All authors declare that they have no conflict of interest.

## Funding

This work was supported by grants from the Instituto de Salud Carlos III, State Secretary of R+D+I of Spain, and FEDER/FSE (FIS 20/1040, 19/319) and grant UM1 CA186107 from National Institutes of Health.

## Ethics statement

The authors certify that they comply with the ethical guidelines for authorship and publishing of the *Journal of Cachexia, Sarcopenia and Muscle*.^42^

## Supporting information




**Table S1.** Relative risks (95% CI) of frailty additionally adjusted for physical activity according to quintiles of protein intake (% of energy) among 85,871 women aged ≥60y in the Nurses' Health Study.
**Table S2.** Relative risks (95% CI) of frailty according to protein intake (% of energy), stratified by lifestyle factors among 85,871 women aged ≥60y in the Nurses' Health Study.
**Table S3.** Relative risks (95% CI) of frailty according to quintiles of protein intake (% of energy) among 69,441 women without any frailty criteria at baseline, aged ≥60y in the Nurses' Health Study.
**Table S4.** Relative risks (95% CI) of a stricter definition of frailty requiring ≥4 criteria according to quintiles of protein intake (% of energy) among 87,247 women aged ≥60y in the Nurses' Health Study.
**Table S5.** Relative risks (95% CI) of frailty according to quintiles of protein intake (% of energy) among 64,436 women aged ≥60y in the Nurses' Health Study, 8 year lagged analysis.
**Table S6**. Relative risks (95% CI) of frailty according to quintiles of the most recent protein intake (% of energy) among 65,236 women aged ≥60y in the Nurses' Health Study.
