# Supplementary material for: Protein intake and risk of frailty among older women in the Nurses' Health Study
Source: J Cachexia Sarcopenia Muscle. 2022 Mar 23;13(3):1752–61. doi: 10.1002/jcsm.12972 (PMC9178161; doi:10.1002/jcsm.12972)
Supplement: Supplementary file 1 — Table S1. Relative risks (95% CI) of frailty additionally adjusted for physical activity according to quintiles of protein intake (% of energy) among 85,871 women aged ≥60y in the Nurses' Health Study. Table S2. Relative risks (95% CI) of frailty according to protein intake (% of energy), stratified by lifestyle factors among 85,871 women aged ≥60y in the Nurses' Health Study. Table S3. Relative risks (95% CI) of frailty according to quintiles of protein intake (% of energy) among 69,441 women without any frailty criteria at baseline, aged ≥60y in the Nurses' Health Study. Table S4. Relative risks (95% CI) of a stricter definition of frailty requiring ≥4 criteria according to quintiles of protein intake (% of energy) among 87,247 women aged ≥60y in the Nurses' Health Study. Table S5. Relative risks (95% CI) of frailty according to quintiles of protein intake (% of energy) among 64,436 women aged ≥60y in the Nurses' Health Study, 8 year lagged analysis. Table S6. Relative risks (95% CI) of frailty according to quintiles of the most recent protein intake (% of energy) among 65,236 women aged ≥60y in the Nurses' Health Study. [file JCSM-13-1752-s001.pdf]

Supporting information

Protein intake and risk of frailty among older women in the Nurses´ Health Study

Ellen A. Struijk, Teresa T. Fung, Fernando Rodriguez-Artalejo, Heike A. Bischoff-Ferrari, Frank B. Hu, Walter C. Willett, Esther Lopez-Garcia.

Supplemental table 1. Relative risks (95% CI) of frailty additionally adjusted for physical activity according to quintiles of protein intake (% of energy) among 85,871 women aged ≥60y in the Nurses' Health Study.

	Q1	Q2	Q3	Q4	Q5	P for trend
**Total protein**						
Multivariable model	1.00	0.97 (0.92; 1.03)	0.99 (0.93; 1.05)	0.98 (0.92; 1.04)	1.07 (1.01; 1.15)	0.02
**Plant protein**						
Multivariable model	1.00	0.93 (0.89; 1.00)	0.90 (0.85; 0.96)	0.87 (0.82; 0.93)	0.87 (0.81; 0.93)	<0.001
**Animal protein**						
Multivariable model	1.00	0.99 (0.93; 1.05)	1.00 (0.94; 1.06)	1.01 (0.95; 1.07)	1.08 (1.01; 1.15)	0.01
**Dairy protein**						
Multivariable model	1.00	1.01 (0.96; 1.07)	1.01 (0.96; 1.07)	1.05 (0.99; 1.11)	1.04 (0.98; 1.10)	0.15

Adjusted for: age (months), calendar time (4-y interval), body mass index (<25.0, 25.0-29.9, ≥30.0 kg/m^2), smoking status (never, past, and current 1-14, 15-24, and ≥25 cigarettes/day), alcohol intake (0, 1.0-4.9, 5.0-14.9, or ≥15.0 g/d), energy intake (quintiles of kcal/d) and medication use (aspirin, postmenopausal hormone therapy, diuretics, β-blockers, calcium channel blockers, ACE inhibitors, other blood pressure medication, statins and other cholesterol lowering drugs, insulin, oral hypoglycemic medication), percentages of energy from saturated fat, monounsaturated fat, polyunsaturated fat, *trans* fat and dietary cholesterol (all in quintiles), the Alternate Healthy Eating Index (quartiles) and physical activity (quintiles).
*Plant protein models are adjusted for animal protein and vice versa. Dairy protein models are adjusted for non-dairy animal protein and plant protein.

Supplemental table 2 Relative risks (95% CI) of frailty according to protein intake (% of energy), stratified by lifestyle factors among 85,871 women aged ≥60y in the Nurses' Health Study.

	Total protein	P interaction	Plant protein	P interaction	Animal protein	P interaction	Dairy protein	P interaction
BMI								
<25 kg/m²	1.01 (0.94; 1.09)	0.004	0.73 (0.57; 0.92)	0.89	1.03 (0.95; 1.11)	0.01	1.02 (0.91; 1.13)	0.92
≥25 kg/m²	1.10 (1.04; 1.16)		0.75 (0.62; 0.90)		1.10 (1.04; 1.16)		1.06 (0.98; 1.15)	
Physical activity								
Below median	1.03 (0.97; 1.09)	0.99	0.71 (0.59; 0.86)	0.28	1.04 (0.98; 1.10)	0.74	1.01 (0.93; 1.09)	0.64
Above median	1.10 (1.03; 1.19)		0.79 (0.63; 1.00)		1.11 (1.03; 1.19)		1.10 (0.99; 1.22)	
AHEI score								
Below median	0.99 (0.93; 1.05)	0.08	0.53 (0.43; 0.64)	0.03	1.01 (0.95; 1.07)	0.30	0.97 (0.89; 1.05)	0.15
Above median	1.03 (0.97; 1.10)		0.71 (0.58; 0.86)		1.04 (0.98; 1.11)		1.04 (0.94; 1.15)	
Baseline age								
<70	1.03 (0.94; 1.12)	0.08	0.76 (0.56; 1.01)	0.26	1.04 (0.96; 1.14)	0.05	1.03 (0.90; 1.17)	0.25
≥70	1.05 (1.00; 1.11)		0.73 (0.62; 0.86)		1.05 (1.00; 1.11)		1.03 (0.96; 1.11)	

[1] Adjusted for: age (months), calendar time (4-y interval), body mass index (<25.0, 25.0-29.9, ≥30.0 kg/m²), smoking status (never, past, and current 1-14, 15-24, and ≥25 cigarettes/day), alcohol intake (0, 1.0-4.9, 5.0-14.9, or ≥15.0 g/d), energy intake (quintiles of kcal/d) and medication use (aspirin, postmenopausal hormone therapy, diuretics, β-blockers, calcium channel blockers, ACE inhibitors, other blood pressure medication, statins and other cholesterol lowering drugs, insulin, oral hypoglycemic medication), percentages of energy from saturated fat, monounsaturated fat, polyunsaturated fat, *trans* fat and dietary cholesterol (all in quintiles), and the Alternate Healthy Eating Index (quartiles).

*Plant protein models are adjusted for animal protein and vice versa. Dairy protein models are adjusted for non-dairy animal protein and plant protein.

Supplemental table 3. Relative risks (95% CI) of frailty according to quintiles of protein intake (% of energy) among 69,441 women without any frailty criteria at baseline, aged ≥60y in the Nurses' Health Study.

	Q1	Q2	Q3	Q4	Q5	P for trend
**Total protein**						
Multivariable model	1.00	0.92 (0.86; 0.99)	0.97 (0.90; 1.03)	0.94 (0.88; 1.01)	1.06 (0.98; 1.15)	0.07
**Plant protein**						
Multivariable model	1.00	0.93 (0.87; 0.99)	0.91 (0.85; 0.97)	0.86 (0.79; 0.92)	0.85 (0.78; 0.92)	<0.001
**Animal protein**						
Multivariable model	1.00	0.98 (0.91; 1.05)	0.97 (0.90; 1.04)	0.99 (0.92; 1.07)	1.06 (0.98; 1.15)	0.07
**Dairy protein**						
Multivariable model	1.00	0.98 (0.92; 1.05)	1.00 (0.93; 1.07)	1.02 (0.95; 1.09)	1.01 (0.93; 1.08)	0.60

Adjusted for: age (months), calendar time (4-y interval), body mass index (<25.0, 25.0-29.9, ≥30.0 kg/m^2), smoking status (never, past, and current 1-14, 15-24, and ≥25 cigarettes/day), alcohol intake (0, 1.0-4.9, 5.0-14.9, or ≥15.0 g/d), energy intake (quintiles of kcal/d) and medication use (aspirin, postmenopausal hormone therapy, diuretics, β-blockers, calcium channel blockers, ACE inhibitors, other blood pressure medication, statins and other cholesterol lowering drugs, insulin, oral hypoglycemic medication), percentages of energy from saturated fat, monounsaturated fat, polyunsaturated fat, *trans* fat and dietary cholesterol (all in quintiles), and the Alternate Healthy Eating Index (quartiles).

*Plant protein models are adjusted for animal protein and vice versa. Dairy protein models are adjusted for non-dairy animal protein and plant protein.

Supplemental table 4. Relative risks (95% CI) of a stricter definition of frailty requiring ≥4 criteria according to quintiles of protein intake (% of energy) among 87,247 women aged ≥60y in the Nurses' Health Study.

	Q1	Q2	Q3	Q4	Q5	P for trend
**Total protein**						
Multivariable model	1.00	0.99 (0.88; 1.10)	1.07 (0.95; 1.19)	1.02 (0.91; 1.14)	1.08 (0.95; 1.22)	0.20
**Plant protein**						
Multivariable model	1.00	0.96 (0.86; 1.06)	0.80 (0.71; 0.89)	0.87 (0.77; 0.98)	0.86 (0.75; 0.98)	0.01
**Animal protein**						
Multivariable model	1.00	1.01 (0.90; 1.13)	1.05 (0.94; 1.18)	1.08 (0.96; 1.21)	1.07 (0.94; 1.21)	0.20
**Dairy protein**						
Multivariable model	1.00	0.91 (0.82; 1.01)	1.01 (0.90; 1.12)	1.01 (0.91; 1.13)	1.01 (0.90; 1.13)	0.35

Adjusted for: age (months), calendar time (4-y interval), body mass index (<25.0, 25.0-29.9, ≥30.0 kg/m^2), smoking status (never, past, and current 1-14, 15-24, and ≥25 cigarettes/day), alcohol intake (0, 1.0-4.9, 5.0-14.9, or ≥15.0 g/d), energy intake (quintiles of kcal/d) and medication use (aspirin, postmenopausal hormone therapy, diuretics, β-blockers, calcium channel blockers, ACE inhibitors, other blood pressure medication, statins and other cholesterol lowering drugs, insulin, oral hypoglycemic medication), percentages of energy from saturated fat, monounsaturated fat, polyunsaturated fat, *trans* fat and dietary cholesterol (all in quintiles), and the Alternate Healthy Eating Index (quartiles).
*Plant protein models are adjusted for animal protein and vice versa. Dairy protein models are adjusted for non-dairy animal protein and plant protein.

Supplemental table 5. Relative risks (95% CI) of frailty according to quintiles of protein intake (% of energy) among 64,436 women aged ≥60y in the Nurses' Health Study, 8 year lagged analysis.

	Q1	Q2	Q3	Q4	Q5	P for trend
**Total protein**						
Multivariable model	1.00	1.01 (0.94; 1.07)	1.02 (0.95; 1.09)	0.99 (0.93 1.07)	1.15 (1.06; 1.23)	<0.001
**Plant protein**						
Multivariable model	1.00	0.98 (0.92; 1.04)	0.91 (0.85; 0.97)	0.95 (0.88; 1.01)	0.96 (0.89; 1.04)	0.26
**Animal protein**						
Multivariable model	1.00	1.03 (0.96; 1.10)	1.02 (0.96; 1.09)	1.00 (0.93; 1.07)	1.14 (1.06; 1.23)	0.002
**Dairy protein**						
Multivariable model	1.00	0.97 (0.91; 1.03)	0.97 (0.91; 1.03)	0.99 (0.93; 1.06)	1.03 (0.97; 1.11)	0.16

Adjusted for: age (months), calendar time (4-y interval), body mass index (<25.0, 25.0-29.9, ≥30.0 kg/m^2), smoking status (never, past, and current 1-14, 15-24, and ≥25 cigarettes/day), alcohol intake (0, 1.0-4.9, 5.0-14.9, or ≥15.0 g/d), energy intake (quintiles of kcal/d) and medication use (aspirin, postmenopausal hormone therapy, diuretics, β-blockers, calcium channel blockers, ACE inhibitors, other blood pressure medication, statins and other cholesterol lowering drugs, insulin, oral hypoglycemic medication), percentages of energy from saturated fat, monounsaturated fat, polyunsaturated fat, *trans* fat and dietary cholesterol (all in quintiles), and the Alternate Healthy Eating Index (quartiles).
*Plant protein models are adjusted for animal protein and vice versa. Dairy protein models are adjusted for non-dairy animal protein and plant protein.

Supplemental table 6. Relative risks (95% CI) of frailty according to quintiles of the most recent protein intake (% of energy) among 65,236 women aged ≥60y in the Nurses' Health Study.

	Q1	Q2	Q3	Q4	Q5	P for trend
**Total protein**						
Multivariable model	1.00	0.90 (0.84 0.96)	0.93 (0.87; 0.99)	0.91 (0.85; 0.97)	0.89 (0.83; 0.95)	0.003
**Plant protein**						
Multivariable model	1.00	0.90 (0.85; 0.95)	0.78 (0.73; 0.83)	0.78 (0.72; 0.83)	0.75 (0.69; 0.80)	<0.001
**Animal protein**						
Multivariable model	1.00	0.97 (0.91; 1.03)	0.93 (0.87; 1.00)	0.94 (0.88; 1.01)	0.91 (0.85; 0.98)	0.01
**Dairy protein**						
Multivariable model	1.00	0.93 (0.86; 0.99)	0.93 (0.87; 1.00)	0.92 (0.86; 0.99)	0.96 (0.90 1.03)	0.55

Adjusted for: age (months), calendar time (4-y interval), body mass index (<25.0, 25.0-29.9, ≥30.0 kg/m^2), smoking status (never, past, and current 1-14, 15-24, and ≥25 cigarettes/day), alcohol intake (0, 1.0-4.9, 5.0-14.9, or ≥15.0 g/d), energy intake (quintiles of kcal/d) and medication use (aspirin, postmenopausal hormone therapy, diuretics, β-blockers, calcium channel blockers, ACE inhibitors, other blood pressure medication, statins and other cholesterol lowering drugs, insulin, oral hypoglycemic medication), percentages of energy from saturated fat, monounsaturated fat, polyunsaturated fat, *trans* fat and dietary cholesterol (all in quintiles), and the Alternate Healthy Eating Index (quartiles).

*Plant protein models are adjusted for animal protein and vice versa. Dairy protein models are adjusted for non-dairy animal protein and plant protein .
